# Successful Arthrodesis Using a Blended Allograft and Autograft Mixture in Lumbar Interbody Fusion: A Retrospective Case Series

**DOI:** 10.7759/cureus.69476

**Published:** 2024-09-15

**Authors:** Jay Fiechter, Anthony N Baumann, Micah Smith

**Affiliations:** 1 Department of Orthopedics, Indiana University School of Medicine, Fort Wayne, USA; 2 Department of Rehabilitation Services, University Hospitals, Cleveland, USA; 3 Department of Orthopedics, Orthopaedics Northeast, Fort Wayne, USA

**Keywords:** allograft, arthrodesis, fusion, orthopedics, spine surgery

## Abstract

Introduction

Achieving successful arthrodesis after lumbar interbody fusion remains a challenge, especially for minimally invasive surgical approaches that limit the amount of local bone autograft. However, using an allograft blend as an autograft extension mixture may hold promise but requires further research. The purpose of this study is to examine the impact of an allograft blend added to autograft on the quality of arthrodesis after lumbar interbody fusion in adult patients.

Methods

This study is a retrospective case series of adult patients (>21 years old) who underwent lumbar interbody fusion between October 2021 and January 2022, performed by a single spine surgeon. The quality of arthrodesis was assessed via the Bridwell grade (I-IV) for up to six months. The impact of surgical technique, age, sex, or amount of allograft utilized during fusion on Bridwell grade was assessed.

Results

Patients (n = 18; 27 levels fused) had a mean age of 58.6 (1.9) years and a mean BMI of 32.8 (1.2) kilograms per meter squared (kg/m²). A mean of 18.0 (standard deviation = 2.4) cubic centimeters (cc) (range: 3.4-50.0 cc) of allograft was used per fused level. A Bridwell grade of I (successful arthrodesis) was achieved at three months in 11.1% (3/27) of fusions and at six months in 85.2% (23/27) of fusions. Four fusions remained at a Bridwell grade of II at six months and subsequently achieved complete arthrodesis at 12 months. No patients received a Bridwell grade of IV (lucency with collapse of graft) at three- or six-month follow-up. There was no difference in Bridwell grade when stratified by surgical technique, age, sex, or amount of allograft used.

Conclusion

The allograft and autograft blend utilized in this study resulted in successful arthrodesis at all fused levels after one year, irrespective of surgical technique or other patient factors. Prospective studies with larger sample sizes are needed to corroborate the findings of this small case series.

## Introduction

Lumbar joint pathology, including degenerative disc disease (DDD), is an age-associated patient morbidity that presents a significant treatment challenge for spine surgeons [1-4]. Conservative treatment (i.e., treatment not including surgery) is pursued first for DDD and may include pain medication, anti-inflammatory medications, injections, and physical therapy. If conservative treatment fails to improve symptoms related to lumbar DDD, routine surgical interventions, such as lumbar interbody fusion, can be considered to improve stability in the degenerated portion of the spine and decrease symptoms [5]. During a typical lumbar interbody fusion, the diseased disc is often removed, the endplates are then prepared appropriately, followed by the placement of an interbody spacer, and the remaining space is finally filled with graft material to promote arthrodesis of the two adjacent vertebrae. To facilitate arthrodesis, any bone removed during the spinal operation is generally cleaned and processed for use as autograft material at the fusion site. Over the years, many different approaches and types of lumbar interbody fusion have been examined in the literature, such as anterior lumbar interbody fusion (ALIF) [6,7], open transforaminal lumbar interbody fusion (TLIF) [8,9], and minimally invasive trans-kambin lumbar interbody fusion (MIS TKLIF) [10-12], each with its own proposed benefits and drawbacks.

One drawback of minimally invasive approaches to lumbar interbody fusion is that insufficient amounts of local bone may be harvested from the operating site [1], limiting the amount of autograft available and threatening proper arthrodesis. Indeed, rates of pseudoarthrosis after lumbar fusion may range from 5% to 15% [13], representing a significant challenge that has not yet been overcome by existing surgical techniques [14]. One method to address this limitation is to make an additional incision and harvest autograft from the patient’s iliac crest, although this may lead to additional adverse events for the patient [15,16]. A counteracting strategy is to use allograft for lumbar fusion, which also offers unique benefits and drawbacks but often results in similar fusion rates when compared to autograft utilization [17-19]. Based on this information, it is possible that a blend of both autograft and allograft materials, in order to reap the benefits of both methods of arthrodesis, may provide an ideal environment for vertebral fusion. This study aims to increase the small pool of research on this topic. Therefore, the purpose of this study is to examine the impact of a 1:1 blend of bone morphogenic protein, cortical fiber allograft, and synthetic quad-phasic bone void filler added to the intraprocedural harvest autograft as an extension mixture on rates of arthrodesis after lumbar interbody fusion in adult patients, in order to investigate novel methods to improve arthrodesis rates and enhance surgical decision-making.

## Materials and methods

Study design

This is a retrospective case series of adult patients (>21 years old) who underwent lumbar interbody fusion with a 1:1 allograft blend combined with harvested autograft performed by a single fellowship-trained spine orthopedic surgeon between October 2021 and January 2022 at a single institution. This study was approved by the Parkview Health Institutional Review Board (ONE22-0314). 

Inclusion criteria

Inclusion criteria were adult patients (>21 years old), a diagnosis of degenerative lumbar pathology (DDD, spondylolisthesis, or lumbar spinal stenosis), the use of a lumbar interbody fusion technique of any approach (ALIF, MIS TKLIF, TLIF), and the use of a 1:1 allograft blend combined with autograft.

Exclusion criteria

Exclusion criteria were patients younger than 21 years old; spinal trauma or spinal fractures as an indication for lumbar interbody fusion; infection; active malignancy; pregnancy; lack of adequate follow-up (at least six months); and use of a graft other than the allograft or autograft combination.

Procedure specifics

Each patient underwent iliac autograft harvest via a core reamer, in which the surgeon utilized one point of entry on the iliac crest and performed three to four passes, while fanning posteriorly and anteriorly. The goal was to harvest about 5-10 cubic centimeters (cc) of autograft material from each patient to be combined with the 1:1 allograft blend. This autograft material was then mixed with the 1:1 allograft blend to increase the total volume of graft material. In terms of surgical methods (ALIF versus open TLIF versus MIS TKLIF), the surgical approach was at the discretion of the surgeon, who determined the most appropriate surgery for each patient based on their symptoms, risk factors, and medical history.

Data collection

Data collected included patient demographics, comorbidities, body mass index (BMI), diagnoses, type of surgery performed, and number/location of fused levels in the lumbar spine. Two-week, six-week, three-month, and six-month follow-up radiographs were graded by a board-certified, fellowship-trained spine surgeon using the Bridwell Anterior Fusion Grading System (Table 1). For levels that were not fused by six months, those levels were followed up to one year or until achieving grade I complete fusion. Grading was performed by the operating surgeon in this study. Upright radiographs assessed included anterior-to-posterior, lateral, spot lateral, flexion, and extension films for each patient at each follow-up interval. The radiographic series at each follow-up appointment were assessed by the surgeon, and a single compiled grade from the Bridwell system was recorded for each fusion level at each follow-up time period.

**Table 1:** Grades and corresponding descriptions for the Bridwell system for arthrodesis

Bridwell grade	Description
I	Fused with remodeling and trabeculae present
II	Graft intact, not fully remodeled and incorporated, but no lucency present
III	Graft intact, but a definite lucency at the top or bottom of the graft
IV	Definitely not fused with resorption of bone graft and with collapse

Statistical analysis

Differences between the mean fusion grade at varying follow-ups were statistically analyzed using the Kruskal-Wallis test due to the small sample size and multiple comparisons. Bridwell grades at each follow-up were compared using the Kruskal-Wallis test. The two-week radiograph grades were analyzed against the six-week grades; the six-week grades were analyzed against the three-month grades; and the three-month grades were analyzed against the six-month grades. Furthermore, the mean Bridwell grades at each follow-up were also assessed by stratifying by type of surgery, age, sex, BMI, quantity of allograft blend, and presence or absence of diabetes. The mean Bridwell grade, with standard deviations, was calculated in Excel (Microsoft® Corp., Redmond, WA, USA) for Mac 2022 for each group at each radiographic interval. The free Kruskal-Wallis calculator at socscistatistics.com was utilized to perform all other statistical calculations.

## Results

Patient demographics

Patients (n = 18; 27 levels fused) had a mean age of 58.6 (standard deviation = 1.9) years (range: 37-79 years) and a mean BMI of 32.8 (1.2) kilograms per meter squared (kg/m²) (range: 23.2-40.3 kg/m²). Of the 27 interbody fusion levels, 14 levels (51.9%) were done in males and 13 levels (48.1%) were done in females, with a mean of 18.0 (2.4) cc (range: 3.4-50.0 cc) of allograft used per fused level. Six levels (22.2%) were fused in the presence of diabetes, and three levels (11.1%) were fused in the presence of active smoking (Table 2). Of the 27 fused levels, one level (3.7%) was fused via ALIF, 10 levels (37.0%) were fused via open TLIF, and 16 levels (59.3%) were fused via MIS TLIF. In terms of lumbar pathology, 42.1% of patients (n = 8) had lumbar anterolisthesis, 100.0% of patients (n = 18) had lumbar stenosis, and 50.0% of patients (n = 9) had a history of prior lumbar surgery.

**Table 2:** Patient demographics for the current study Data includes the number of patients, number of fused levels, age, male and female distribution, body mass index, specifics on the levels fused, surgical approaches, and patient comorbidities. SD, standard deviation; MIS TKLIF, minimally invasive trans-kambin lumbar interbody fusion; TLIF, transforaminal lumbar interbody fusion; ALIF, anterior lumbar interbody fusion

Patient demographics	
Patients, n (fused levels)	18 (27)
Patient age, mean (SD)	58.6 (1.8)
Male fused levels, n (%)	14 (51.9%)
Female fused levels, n (%)	13 (48.1%)
Body mass index, mean (SD)	32.2 (1.2)
Level fused, n (%)	
L1/L2	3 (11.1%)
L2/L3	6 (22.2%)
L3/L4	6 (22.2%)
L4/L5	9 (33.3%)
L5/S1	3 (11.1%)
Surgical approach, n (%)	
MIS TKLIF	16 (59.3%)
Open TLIF	10 (37.0%)
ALIF	1 (3.7%)
Comorbidities, n (%)	
Diabetes	6 (22.2%)
Osteoporosis	2 (7.4%)
Renal disease	4 (14.8%)
Cancer	0 (0.0%)
Active smoker	3 (11.1%)

Arthrodesis outcomes

Complete arthrodesis (Bridwell grade I) was seen in 11.1% (n = 3 levels) of fusion levels at three months and 85.2% (n = 23 levels) at six months after lumbar interbody fusion. At six months after surgery, the remaining four fusions (14.8%) had a Bridwell grade II arthrodesis and were evaluated later to have grade I complete arthrodesis by 12 months. Importantly, none (0.0%) of the fused levels regressed to a Bridwell grade IV arthrodesis with collapse of the graft material at three months or six months after lumbar interbody fusion. There was a statistically significant difference between the mean three-month and six-month Bridwell grades in comparison to the two-week and six-week follow-ups (p < 0.0001 and p < 0.0001, respectively). There was no statistically significant difference between the mean Bridwell grade at the six-week follow-up and the two-week follow-up (p = 0.2389).

Arthrodesis outcomes stratified by variables

There was no statistically significant difference in mean Bridwell grade when stratified by surgical approach (open TLIF versus MIS TKLIF; p = 0.7122), age (<65 years old versus ≥65 years old; p = 0.0965), sex (male versus female; p = 0.4817), BMI (<30 kg/m² versus ≥30 kg/m²; p = 0.3545), allograft amount (≤15 cc versus >15 cc; p = 0.4701), or diabetes (presence versus absence; p = 0.6934) at six-month follow-up. While none of these stratifications found statistical significance, it is highly likely that these comparisons were underpowered due to the small sample size.

## Discussion

This retrospective case series utilized a mix of two allograft products in combination with intraoperatively harvested autograft to increase graft quantity and promote arthrodesis in lumbar interbody fusion, aiming to demonstrate improved arthrodesis rates among adult patients. Given that pseudoarthrosis can occur in 5-15% of patients undergoing lumbar fusion [13], it is notable that 100.0% of patients (n = 18) had complete arthrodesis (Bridwell grade I) at all fused levels (n = 27) at 12-month follow-up, with 85.2% of fused levels (n = 23) achieving complete arthrodesis at six months. Given that the literature has shown similar rates of fusion when comparing allograft versus autograft [17-19], the results of this study cautiously hint at the possibility that the unique 1:1 allograft blend combined with autograft may lead to improved arthrodesis rates. This is important as pseudoarthrosis can be an indication for reoperation, which can lead to increased patient complication rates [20]. Importantly, none of the levels included in this study received a Bridwell grade IV, which is defined as “definitely not fused, with resorption of bone graft and collapse.” Although this case series cannot prove the superiority of the allograft and autograft combination over conventional graft choices due to the lack of a control group, small sample size, and retrospective study design, the findings of this study may spur future research into optimal graft compositions and add to the growing body of research on autograft extenders. Additional research on this topic hopefully aid spine surgeons in achieving complete arthrodesis and lead to improved patient outcomes [19].

The importance of this study relies on the description of the utilized allograft and autograft blend, as well as the biological rationale for the very high arthrodesis rates seen in this study after lumbar interbody fusion via various approaches. Successful graft material must possess the capability to be both osteogenic, osteoinductive, and osteoconductive, commonly referred to as the pillars of bone regeneration [21]. When autografts are traditionally harvested in real time from the patient, the graft possesses all three pillars of bone regeneration and offers no risk of rejection, as it represents the patient’s own tissue. In contrast, allograft materials are processed to reduce the risk of rejection, increasing the safety of using allograft during lumbar interbody fusion. Unfortunately, this process typically destroys the osteoinductive pillar of bone regeneration, as well as the mesenchymal stem cells required for the osteogenic pillar of bone regeneration [22]. However, as mentioned previously, a surgeon may be forced to use allograft materials if they are unable to harvest enough bone from the surgical site or do not wish to create another incision to harvest bone from another site on the patient’s body. Therefore, this study used an allograft blend made from two different types of allograft. The first, bone morphogenic allograft, is a unique material that actually possesses both osteoinductive and osteoconductive properties, contrary to other allograft materials. The second allograft material is a synthetic quad phasic bone void filler that contains multiple ingredients with different resorption profiles, offering faster bone regrowth in comparison to traditional bioglass bone fillers. Additionally, this second allograft material also exhibits nanotopography on each particle, increasing surface area to interact with osteocytes and promote vascularization and bone growth [23]. Importantly, the use of both allografts in this manner was off-label.

As the primary outcome in this study is arthrodesis, as described by the Bridwell grading scale, it is important to examine this scale and its utility in assessing arthrodesis after lumbar fusion. The Bridwell grading scale was created in 1995 and contains four categories to assess fusion quality [24,25]. Since 1995, this grading scale has been used by many studies and is an important part of the literature in assessing arthrodesis. A recent study analyzed many grading systems, including the Bridwell system, and found reasonable intra-observer correlation [25]. However, the diagnostic accuracy of the Bridwell system is not likely to be strongly correlated with clinical outcomes and has an overall low diagnostic accuracy [25]. It should be noted that the gold standard for assessing the quality of arthrodesis is surgical exploration; therefore, this study used the Bridwell grading scale as a reasonable and acceptable alternative, given the lack of reoperation. Importantly, none of the included patients (n = 18) required reoperation over a one-year period. While it would have been helpful to have an additional outcome scale, this study only has the Bridwell system to rely upon. Finally, while this study is limited by sample size, it is important to note that none of the factors examined in this study - such as procedure type, age, sex, BMI, amount of allograft, and presence of diabetes - were associated with a change in mean Bridwell grade. However, this is likely due to the underpowered nature of these comparisons, and more research is needed.

Overall, the results of this study need to be understood in the context of its limitations. One limitation is the lack of a control group, which limits direct comparisons on the effectiveness of the allograft and autograft combination blend used in this study. Additionally, with such a small sample size, there were not enough data points to statistically assess the importance of other comorbidities, such as smoking status, renal disease, osteoporosis, and cancer. While the study did include patients with these comorbidities, the number of data points was insufficient for analysis. We also cannot control for the quantity of iliac crest harvested in each individual patient or among the cohort. Furthermore, as stated above, each patient has a different percentage of viable stem cells in the iliac crest autograft, which cannot be controlled for as a confounding variable, thus introducing bias. Additionally, the use of the Bridwell grading system is another limitation, as studies have found discrepancies between grading systems and actual outcomes [25]. However, it remains an acceptable and non-invasive metric utilized by many studies since its initial introduction in 1995 [24,25]. Another limitation is the lack of a blinded clinician to perform the grading, which introduces additional bias. While the surgeon’s intent would be to stay completely objective, a blinded third party would have provided increased assurance of unbiased data. Overall, the findings of this case series, with no cases of pseudoarthrosis after one year, require future prospective studies for collaboration.

## Conclusions

The use of a unique 1:1 allograft blend combined with autograft to promote arthrodesis during lumbar interbody fusion in adult patients may result in improved fusion rates based on a small retrospective case series, as roughly 85% of fused levels were completely fused (Bridwell grade I) at six-month follow-up, with the remaining levels achieving complete fusion at 12-month follow-up. Importantly, none of the fused levels demonstrated fusion failure (Bridwell grade IV), suggesting that this allograft and autograft combination may play a role in lumbar interbody fusion, although this needs to be solidified with future research. The quality of arthrodesis, as determined by the Bridwell scale, was not associated with procedure type, age, sex, presence of diabetes, or amount of allograft, although these comparisons were likely underpowered. Given the concerning rates of pseudoarthrosis in lumbar fusion procedures, future studies should investigate allograft and autograft combination mixtures as a strategy to promote proper arthrodesis.
